# Adapting Coordinated Anxiety Learning and Management for Veterans Affairs Community-Based Outpatient Clinics: Iterative Approach

**DOI:** 10.2196/10277

**Published:** 2018-08-22

**Authors:** Traci H Abraham, Kathy Marchant-Miros, Michael B McCarther, Michelle G Craske, Geoffrey M Curran, Lisa K Kearney, Carolyn Greene, Jan A Lindsay, Michael A Cucciare

**Affiliations:** ^1^ South Central Mental Illness Research, Education, and Clinical Center US Department of Veterans Affairs Little Rock, AR United States; ^2^ Department of Psychiatry University of Arkansas for Medical Sciences Little Rock, AR United States; ^3^ Center for Mental Healthcare and Outcomes Research Central Arkansas Veterans Healthcare System North Little Rock, AR United States; ^4^ Department of Psychology University of California, Los Angles Los Angeles, CA United States; ^5^ Department of Psychiatry and Biobehavioral Sciences University of California, Los Angeles Los Angeles, CA United States; ^6^ Center for Implementation Research University of Arkansas for Medical Sciences Little Rock, AR United States; ^7^ Department of Pharmacy Practice College of Pharmacy University of Arkansas for Medical Sciences Little Rock, AR United States; ^8^ US Department of Veterans Affairs Center for Integrated Healthcare Buffalo, NY United States; ^9^ Department of Psychiatry The University of Texas Health Science Center San Antonio, TX United States; ^10^ Office of Mental Health and Suicide Prevention US Department of Veterans Affairs Washington, DC United States; ^11^ Houston Veterans Affairs Health Services Research & Development Service Center for Innovations in Quality, Effectiveness, and Safety Michael E DeBakey Veterans Affairs Medical Center Houston, TX United States; ^12^ South Central Mental Illness Research, Education, and Clinical Center Houston, TX United States; ^13^ Menninger Department of Psychiatry & Behavioral Sciences Houston, TX United States

**Keywords:** therapy, veterans, depression, anxiety disorders, posttraumatic stress disorder, PTSD

## Abstract

**Background:**

A national priority at the US Department of Veterans Affairs (VA) is to increase the availability and accessibility of evidence-based psychotherapies (EBPs) across all VA medical facilities. Yet many veterans, particularly those who use remote outpatient VA clinics, still do not receive much needed evidence-based treatment. Strategies are needed for supporting mental health providers at rural VA community-based outpatient clinics (CBOCs) as they translate their clinical training to routine practice. The Coordinated Anxiety Learning Management (CALM) program is a computer-delivered program that supports the delivery of cognitive behavioral therapy (CBT) by providers in outpatient settings to patients with depression and anxiety, including posttraumatic stress disorder.

**Objective:**

The objectives of our study were to (1) adapt an existing computer-based program to rural VA CBOCs through feedback from key stakeholder focus groups; (2) develop a prototype of the adapted program; and (3) determine the adapted program’s acceptability and feasibility. Mental health stakeholders included VA leaders (n=4) in the implementation of EBPs, VA experts (n=4) in CBT, VA CBOC mental health providers (n=8), and veterans (n=8) diagnosed with a mental health condition treated using the CALM program and receiving treatment in a VA CBOC.

**Methods:**

An iterative approach comprising 3 waves of focus group discussions was used to develop a modified prototype of CALM. Following each wave of focus group discussions, template analysis was used to rapidly communicate stakeholder recommendations and feedback to the design team. The original program was first adapted through a process of data collection, design modification, and product development. Next, a prototype was developed. Finally, the redesigned program was tested for acceptability and feasibility through a live demonstration.

**Results:**

Key stakeholders suggested modifications to the original CALM program that altered its modules’ appearance by incorporating veteran-centric content. These modifications likely have no impact on the integrity of the original CALM program, but have altered its content to reflect better the demographic characteristics and experiences of rural veterans. Feedback from stakeholder groups indicates that changes will help VA patients identify with the program content, potentially enhancing their treatment engagement.

**Conclusions:**

The development model was effective for economically gathering actionable recommendations from stakeholders to adapt a computer-based program, and it can result in the development of an acceptable and feasible computer-delivered intervention. Results have implications for developing computer-based programs targeting behavior change more broadly and enhancing engagement in EBP.

## Introduction

Ample evidence indicates the effectiveness of evidence-based psychotherapies (EBPs), particularly cognitive behavioral therapy (CBT) [1,2], for treating anxiety and depression, the most common mental health disorders in outpatient health care settings [3-5]. Accordingly, the US Department of Veterans Affairs (VA) has made it a national priority to increase the availability and accessibility of EBPs, particularly CBT, across all VA medical facilities and clinics to veterans needing mental health care [6]. An important component of this strategy is providing intensive, competency-based training in EBPs to VA mental health providers [7].

Despite this effort, broadly implementing CBT and other EBPs in VA treatment settings has been a challenge [8,9], especially at small or remote outpatient clinics, such as most VA community-based outpatient clinics (CBOCs). The challenges inherent in ensuring that EBPs are accessible at rural clinics are evident in results from a study published in 2010, which found that only 1 in 5 veterans with depression, anxiety, or posttraumatic stress disorder (PTSD) received at least one session of psychotherapy and that rural veterans, who are often treated in CBOCs, were even less likely than their urban counterparts to have received any psychotherapy [10]. Barriers to ensuring access to EBPs in VA CBOCs are complex, likely including practitioner factors (eg, resistance to change), training methods used, characteristics of the intervention, and organization or system factors [11]. Although evidence suggests that the disparity found in 2010 has since decreased, rural veterans still receive fewer psychotherapy sessions than their urban peers [12]. Thus, although many providers at VA CBOCs have received training in EBPs, training alone has not been sufficient to ensure that new treatments are translated into routine clinical practice [8,13].

An additional challenge at VA CBOCs is ensuring that treatments are delivered with fidelity (ie, faithfulness to the treatment model). Without adequate fidelity, patients are unlikely to experience optimal outcomes from EBPs, even where they are accessible. Although the VA has made great strides in providing EBP-specific training to providers of mental health care, training alone is not sufficient to ensure fidelity [14,15]. VA CBOCs commonly have only one mental health provider on staff. Providers are, therefore, isolated and unable to consult their peers about difficult cases. Additionally, providers may not have time to take advantage of educational resources that facilitate effective delivery of such EBPs as CBT. Providers who do not perceive themselves as proficient in delivering EBTs are sometimes reluctant to use them with patients [16]. Thus, many providers at small or remote outpatient clinics lack the resources, time, or interactions with colleagues needed to gain proficiency in these skills. Resources that can support and assist CBOC mental health providers in delivering EBPs with fidelity are needed to supplement training and ensure rural veterans’ access to effective mental health treatments.

Over the last decade, the National Institute of Mental Health-funded Coordinated Anxiety Learning Management (CALM) study [17] has addressed similar challenges. The study aimed to implement CBT into non-VA outpatient clinics to support providers with little to no prior training in this treatment. To achieve this goal, researchers developed a computer-delivered program (CALM) facilitating the delivery of CBT with fidelity by mental health providers in outpatient settings [18]. CALM uses a cognitive behavioral framework including psychoeducation, cognitive restructuring, goal setting, exposure, and response prevention. It has the added advantage of being both provider and patient facing, so that patient and provider both look at the computer screen together and proceed through the modules at an individualized pace [17]. The CALM program is clinically effective for a range of anxiety disorders, including panic disorder (PD), generalized anxiety disorder (GAD), and social anxiety disorder (SAD), as well as PTSD and depression [17-19]. It may have the added benefit of helping providers maintain fidelity to the CBT treatment model [20]. Thus, implementation of the CALM program in rural VA outpatient settings may help increase veterans’ receipt of efficacious CBT.

**Figure 1:**
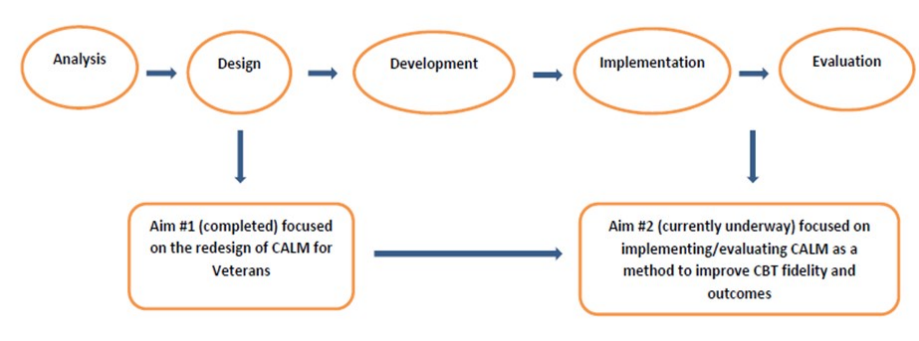
Modified ADDIE (Analysis, Design, Development, Implementation, and Evaluation) model and study aims. CALM: Coordinated Anxiety Learning Management, CBT: cognitive behavioral therapy.

In this study, we sought to adapt the original CALM program for use in VA outpatient settings, thus, supporting VA CBOC mental health providers in delivering CBT to veterans with anxiety, PTSD, and depression. To achieve this objective, we modified a method common to the field of instructional design and technology (IDT) to guide the redesign of the CALM computer program for use in VA CBOCs. IDT models are prescriptive models that describe a set of activities involved in the planning, implementation, and evaluation of instructional programs [21]. Most IDT models share the core elements of analysis, design, development, implementation, and evaluation, applied in an iterative process [22,23]. The Analysis, Design, Development, Implementation, Evaluation model, as seen in Figure 1, an umbrella term referring to these common elements, formed the conceptual basis for the process used to modify the original CALM program in this study [24].

As part of this process, 3 waves of focus group discussions were conducted with 4 groups of key stakeholders to redesign and test the CALM program iteratively for use in VA CBOCs. Stakeholder groups included (1) veterans receiving recent mental health care in a VA CBOC and diagnosed with anxiety disorders and depression, (2) CBOC mental health providers, (3) expert CBT VA clinicians, and (4) VA Central Office leaders with expertise in the implementation of EBPs within VA. In this paper, we describe the iterative process used to redesign and test the modified CALM program, as well as modifications used to adapt the program for use at VA CBOCs.

## Methods

### Recruitment

We recruited participants from 4 key stakeholder groups: veterans (n=11) with a recent (≥1 visit in the prior 6 months) mental health care visit at a VA CBOC and with a diagnosis of one or more of the following: PD, GAD, SAD, PTSD or depression; CBOC mental health providers (n=11) in the southern region of the United States, including nurses, psychiatrists, social workers, and psychologists; VA expert CBT clinicians (n=6); and VA Central Office leaders (n=5) with expertise in implementing EBPs within VA. We conducted 3 waves of focus group discussions with each stakeholder group.

Veteran participants were recruited from one CBOC in Arkansas. Mental health providers were recruited using a general call distributed via email to providers at CBOCs in Arkansas, Louisiana, Texas, and Mississippi. Multiple CBOCs were needed because most CBOCs have only one mental health provider on site. CBT experts were recruited nationally from within the VA, and leaders in implementation of EBPs within VA were recruited from the VA Central Office in Washington, DC CBT experts and leaders in implementation were individually recruited through convenience sampling based on their level of expertise and availability [25].

The purpose of recruiting veteran stakeholders was to help ensure that the modified CALM content was acceptable to the target patient population and reflected veterans’ illness experiences. We included CBOC mental health providers to help ensure that the modified content was acceptable for this group of providers (who serve a largely rural veteran population), images and case studies were appropriate for the target patient population, and the navigation and flow of CALM treatment material met the needs of CBOC providers. Inclusion of VA expert CBT clinicians helped ensure that the empirical support underlying the content of the CALM program was not compromised during the modification process. Finally, inclusion of VA Central Office leaders with expertise in EBP implementation within VA helped the study team ensure that CALM was consistent with prior CBT training efforts within VA.

### Data Collection

Each group of key stakeholders participated in 3 waves of separate focus group discussions. Focus group discussions are a standard method used to garner diverse feedback efficiently on novel and existing products for new product development. CBOC mental health providers, CBT experts, and VA Central Office leaders with expertise in EBP implementation within VA participated in focus group discussions via teleconference with the assistance of Lync, a Web-based meeting portal that allows participants to view the same material simultaneously. In addition to providing feedback via telephone, participants could leave written feedback on the website chat room and email content for the focus group facilitators. Veteran focus groups met in person at a VA CBOC in Hot Springs, Arkansas.

All focus group discussions were co-moderated by the principal investigator (MAC) and a co-investigator (THA). Both researchers have extensive experience in qualitative interviewing and facilitating focus group discussions. An interview guide developed for the study was used to ensure that the discussion remained relatively consistent across stakeholder groups and that groups addressed all relevant topics. The same stakeholders were invited to participate in all 3 waves of focus group discussions to maximize the relevance of feedback (ie, participants knew whether recommendations had been correctly incorporated into the program). Focus group discussions were audiorecorded with permission from participants. The study was approved by the Central Arkansas Veterans Healthcare System Human Research Protections Program.

### Data Analysis

Rapid analytic techniques informed by Sobo et al [26] and Hamilton [27] were used to quickly produce recommendations for modifying the CALM program and economically communicating modifications to the design team. Qualitative data analysts (THA and MAC) first collaborated to develop a prototype summary template in a Word document with three broad domains related to goals of the study: (1) recommendations from focus group participants; (2) evaluative observations or initial reactions or concerns, and (3) questions. They then created categories within each broad domain, reflecting various aspects of the CALM program that would be queried during focus group discussions. A copy of this prototype was then created in a new Word document for each of the 4 focus groups.

To analyze focus group discussions, the lead analyst (THA) listened to the audio recording of the first focus group discussion and systematically populated template categories with data. These data consisted of paraphrased content reflecting stakeholder recommendations, observations, reactions, concerns, and questions. The goal was to capture the full range of responses to questions and comments from focus group discussions. After the lead analyst completed summarizing all content from the focus group discussions in the template, she met with the second analyst (MAC) to discuss analytic findings. Discrepancies in how template content was summarized or categorized were resolved through discussion. This analytic process was repeated for each focus group discussion. For focus group discussions held via teleconference, analysts also incorporated written feedback from the website and emails to facilitators into the template. After analyses were finalized for all 4 focus group discussions, findings were compiled and summarized. Analysts each reviewed the summary to ensure the validity of findings. This analytic process was followed for all 3 waves of focus group discussions.

### Finalizing Recommendations

A panel of experts reviewed the summary template before it was submitted to the design and development team, including study team members with expertise in CBT, the relevant mental health diagnosis, and software development. The purpose of this panel was to prioritize and determine the feasibility of each recommendation (eg, cost and time needed to complete the modification). The summary template was subsequently reduced to actionable recommendations for modification.

### Description of the Original Coordinated Anxiety Learning Management Program

The CALM computerized program was created to guide and train mental health providers in delivering a course of CBT [17]. CALM was not intended, like some other technology-based interventions, to be a self-help intervention (ie, patient facing without provider involvement). Instead, provider and patient use it together, synchronously (ie, it is provider and patient facing). Provider-supported treatments such as CALM have tended to yield enhanced results compared with self-help interventions [28]. A unique feature of the CALM program is that it can be used to treat different anxiety disorders, as well as PTSD and depression. Reductions in symptoms across these conditions are accomplished through the use of basic CBT modules, which are employed across these disorders, coupled with branching modules that are disorder specific [18-20].

## Results

### Wave 1: Wireframe Development

During the first wave of focus group discussions, an overview of CALM was presented to each stakeholder group. This also included screenshots from the program, as well as a detailed description of the functioning of CALM for each disorder. Following the overview, stakeholders were given time to ask questions about CALM; then, they were asked to provide initial reactions or recommendations for modifying and adapting the program for use within VA CBOCs.

#### Summary of Wave 1 Stakeholder Feedback

Recommended modifications to the original program were largely “look and feel” changes pertaining to the images and illustrations in the modules. Stakeholders generally reported that the images and illustrations contained in the original program should be more representative of veterans. They recommended reducing the number of images and illustrations depicting college-aged women and men in white-collar occupations (eg, wearing suits) as well as incorporating imagery better reflecting the gender, age, and economic and ethnic or racial diversity of veteran patients. Veteran stakeholders did not like the background color of the CALM program and overall website template; they wanted a new logo developed specifically for the modified VA program.

Stakeholders generally also recommended modifying the images in the original program to make it more “culturally congruent” because the original program was not designed to acknowledge veterans’ military service. One expert CBT clinician noted that this can be “very invalidating” for some patients. Stakeholders suggested replacing some existing images with images of people in uniform; however, a veteran stakeholder cautioned that while incorporating images of people in uniform, “We don’t need to see a Vet on every single picture we look at.” Stakeholders also suggested using images of individuals with physical limitations in the Behavioral Activation module to encourage veterans with prosthetics or other physical limitations to engage in physical activities.

Furthermore, stakeholders recommended revisions designed to enhance the degree to which veterans could identify with the program content. To achieve this, they suggested incorporating videos into the modules that speak to the unique mental health concerns of veterans. Stakeholders also suggested using existing VA resources, such as the National Center for PTSD’s “About Face” and the VA’s “Make the Connection” websites [28,29]. These websites contain videos in which veterans, family members of veterans, and mental health providers share their personal experiences with mental health concerns and relate individual stories about seeking and receiving help for mental health concerns. They also provide instructions for initiating treatment or seeking immediate help during crises. One CBT expert recommended selecting videos in which multiple veterans shared their experiences to increase the chance that patients will relate to someone in the video. A CBOC mental health provider thought that videos in which veterans describe “service or readjustment issues,” such as reintegration into civilian life, would be particularly useful in helping veterans “connect” with the program content. Stakeholders also recommended incorporating links to existing Web-based psychoeducational materials developed for veterans. Suggested resources included information available at the VA National Center for PTSD and the VA websites. An additional suggestion was to include the telephone number for the national Veterans’ Crisis Line at the end of the Depression Education module.

Finally, stakeholders were generally concerned that some slides seemed too “content heavy” to be engaging. Several expert CBT clinicians and CBOC mental health providers thought that having to read a large amount of material during an hour-long therapy session might be difficult, and expert CBT clinicians were concerned that younger veterans with traumatic brain injuries would be overwhelmed by busy slides. This concern was validated by feedback from veteran stakeholders, who not only recommended reducing the number of words, but removing excessive images and illustrations. One veteran noted: “Pictures: unless it means something, what’s the point?”

#### Wave 1 Modifications to the Coordinated Anxiety Learning Management

**Figure 2:**
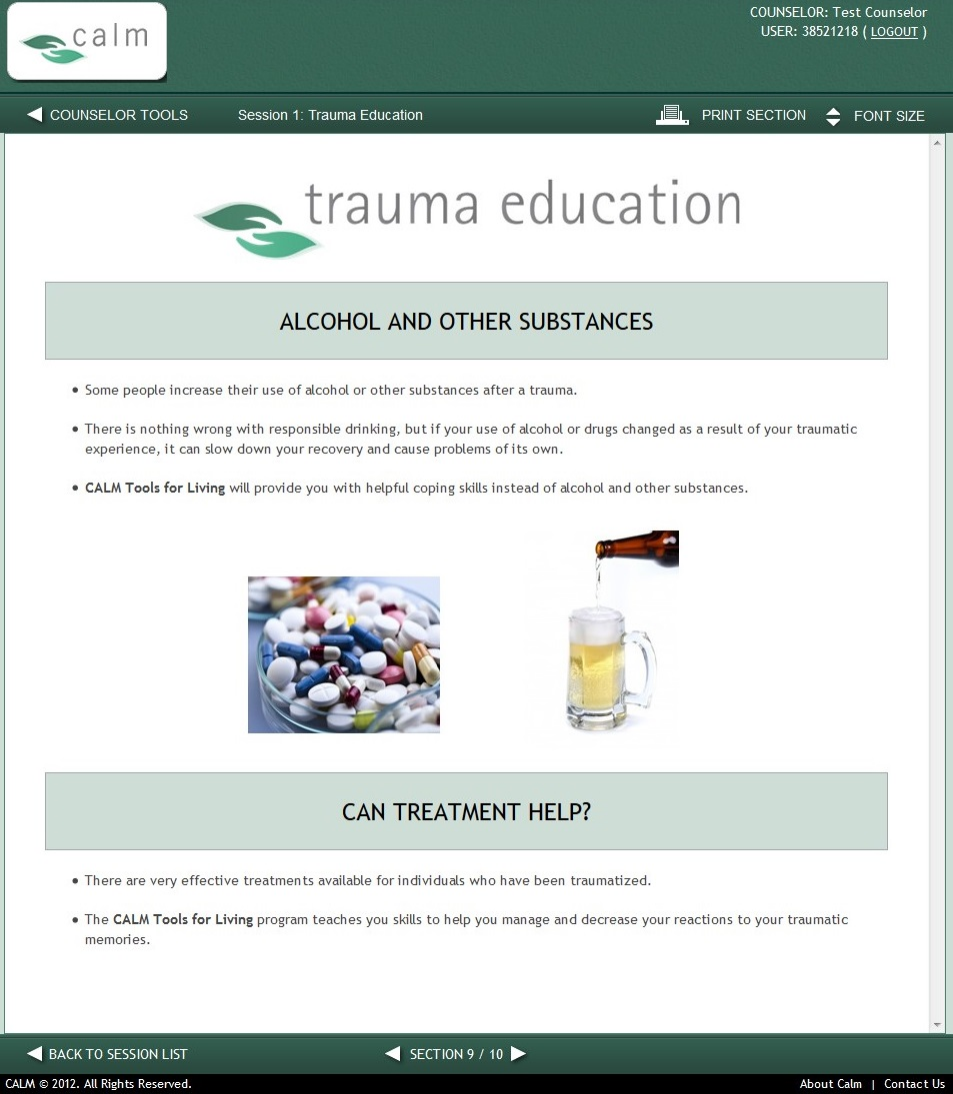
Original Coordinated Anxiety Learning Management (CALM) module content. (Screenshot taken by KMM; Source: University of California, Los Angeles).

**Figure 3:**
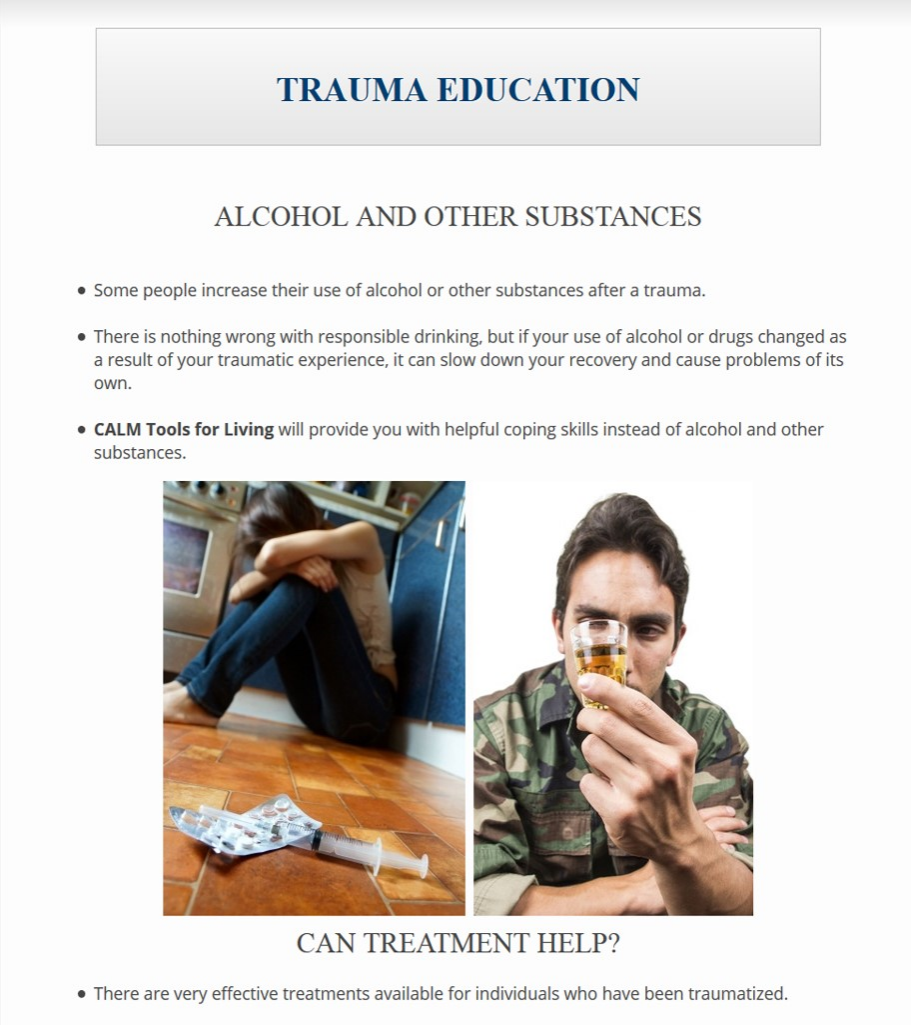
Modified Coordinated Anxiety Learning Management (CALM) module content. (Screenshot taken by KMM; Source: US Department of Veterans Affairs).

A summary of stakeholder recommendations was presented to the expert panel for review, and actionable modifications were identified and prioritized. The study team then collaborated with the software development group to identify images and illustrations better reflecting the gender, age, and economic and ethnic or racial diversity of veterans. They also identified appropriate images of people in military uniform. The images below are examples of CALM module content before (Figure 2) and after modification (Figure 3). The alterations illustrated make use of updated imagery and of veteran stakeholder preferences for color (ie, blue rather than green) and depict an individual in uniform to enhance content relatability.

**Figure 4:**
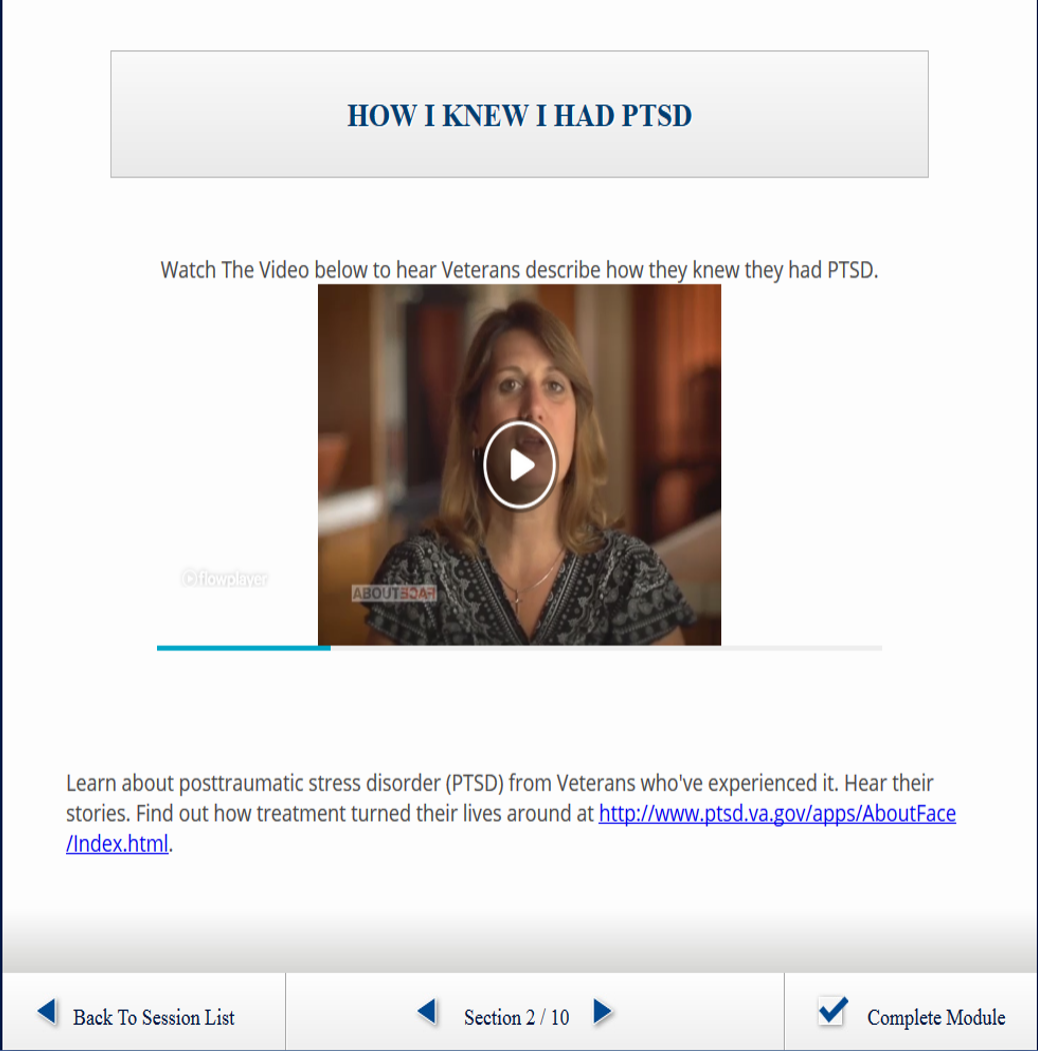
Video featuring veterans describing their experiences with posttraumatic stress disorder (PTSD); used with permission from the National Center for PTSD “About Face” website. (Screenshot taken by KMM; Source: US Department of Veterans Affairs).

The team identified already available Web-based resources with videos of veterans describing their mental health concerns, their experiences with a condition and/or seeking mental health care, and ways that treatment helped them (Figure 4) [29]. This was realistic in the timeframe of the study, which did not allow sufficient time to develop original video content.

Finally, the team followed stakeholder suggestions in identifying links to Web-based resources for veterans and reduced the amount of text on slides identified by stakeholders as too content heavy. These modifications to the original program were submitted to the software development team, which then developed mock-ups, or wireframes, of the initial modifications to show stakeholders in Wave 2.

### Wave 2: Prototype Development

The wireframes were presented during a second wave of focus group discussions with veterans (n=6), CBOC mental health providers (n=10), expert CBT clinicians (n=5), and VA leaders with expertise in the implementation of EBPs within VA (n=5). Wave 2 participants were reminded of the initial version of CALM using graphics and were provided a side-by-side comparison of all modifications. After reviewing the wireframes, stakeholders provided feedback regarding modifications from the first round of focus groups, and a few suggested additional changes to the CALM program.

#### Summary of Wave 2 Stakeholder Feedback

Stakeholders generally agreed that the modified program appeared more “inclusive of veterans that will be using CALM” and “user-friendly” for the veteran population. Although agreeing that the initial modifications were an improvement, one veteran stakeholder requested more images of older males to be more inclusive of Vietnam-era veterans. Stakeholders, particularly veterans, also responded positively to reductions in the amount of text on many slides. One CBOC mental health provider thought that reducing the amount of text would “leave more room for interaction” between providers and veteran patients. Stakeholders also responded positively to the inclusion of videos and links to resources. One younger participant in the veteran focus group who had completed VA treatment for PTSD suggested replacing a video in which veterans described only their treatment experiences with the one in which they explain that treatment is difficult at first, but helps. Finally, one CBOC mental health provider noted that many of his veteran patients were uncomfortable with writing and suggested the program allow veterans the option of verbally describing and recording trauma memories for use in the Exposure module, in addition to writing about them.

#### Wave 2 Modifications to Coordinated Anxiety Learning Management

**Figure 5:**
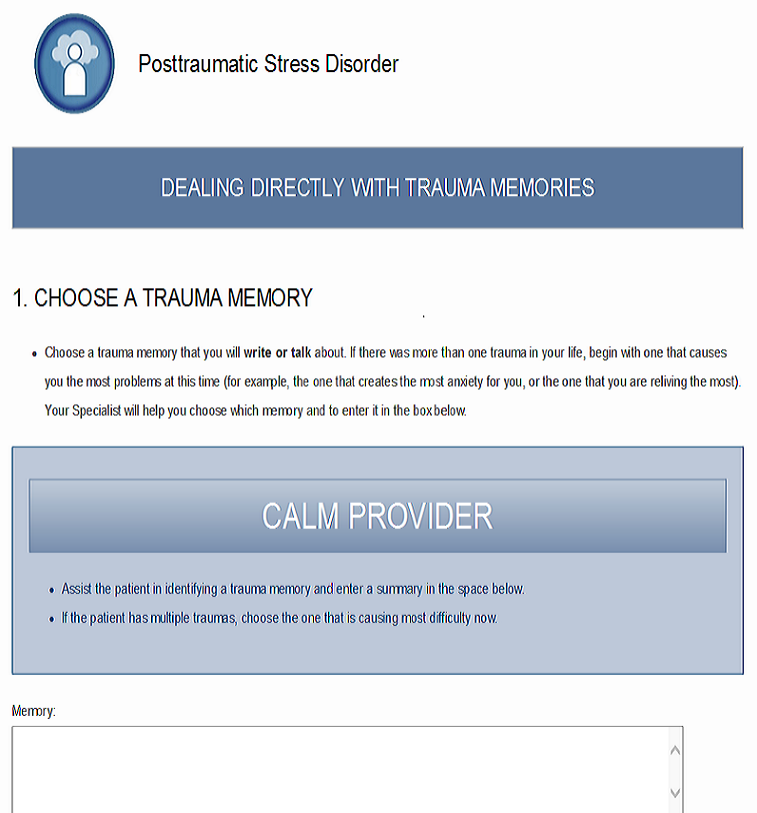
Option allowing veterans to describe and record traumatic experiences. (Screenshot taken by KMM; Source: US Department of Veterans Affairs).

The expert panel again reviewed a summary of recommendations and prioritized actionable modifications from Wave 2 focus group discussions. Following this, the study team identified images and videos per stakeholder recommendations. The principal investigator (MAC) collaborated with one CALM developer (MAC) to incorporate an option allowing veterans who are uncomfortable with writing to verbally describe and record their traumatic experiences (Figure 5). These modifications were submitted to the design team, and a prototype of the modified CALM program was developed.

### Wave 3: Validation of Coordinated Anxiety Learning Management Modifications

During a final wave of focus group discussions, the prototype was pilot-tested by MGC during a live teleconferenced demonstration to 3 stakeholder groups: CBOC mental health providers (n=6), expert CBT clinicians (n=4), and VA leaders in clinic operations and implementation (n=3). Minor problems that arose during the pilot test were recorded in written notes by the study team and rapidly communicated to the design team following the demonstration. Veteran stakeholders (n=4) reviewed modified CALM modules presented in person by the principal investigator (MAC) and looked at an overview of all modifications by the lead qualitative analyst (THA). No remaining concerns about the program were elicited through this final wave of focus groups.

## Discussion

### Principal Findings

In this study, we used an iterative approach to adapt the original CALM program for use within the VA and, particularly, at rural, CBOCs. Overall, key stakeholders suggested modifications to the original CALM program that were largely “look and feel” adaptations that altered the appearance of the modules by incorporating veteran-centric content. This included relatively simple changes, such as replacing images and developing a new template with the VA logo, as well as more significant adaptations, such as embedding videos of veterans describing their treatment and illness experiences and modifying the case studies to better reflect experiences common to rural veterans. These modifications likely have no impact on the integrity of the original CALM program, while altering the content to better reflect the demographic characteristics and experiences of rural veterans.

An additional adaptation was to customize the treatment content to allow veterans the option of orally recounting (as opposed to only writing) their trauma experiences. This modification, along with the overall flexibility of the CALM program to meet the treatment needs of veterans with a wide variety of mental health conditions, is consistent with a long-standing cultural shift at the VA toward the provision of patient-centered health care consisting of a menu of treatment options that can be tailored to each individual veteran’s needs and goals for health and well-being [30,31]. Patient-centered care can improve health outcomes and increase patient satisfaction and self-management of chronic conditions [32,33]. It has also been associated with decreased health care utilization, including the annual number of specialty care visits, less frequent hospitalizations, and fewer laboratory and diagnostic tests [34].

Feedback from stakeholder groups—and, in particular, rural veterans—indicates that these modifications will help VA patients identify with the program content. This could potentially improve patient-provider communication during therapy sessions and increase veteran engagement in EBP for anxiety, depression, and PTSD. Enhancing engagement is important because attrition from EBPs is high among veterans, particularly those with PTSD [35]. In one recent study, of 351 veterans who initiated EBP for PTSD, one third (n=135) dropped out before treatment completion [36]. Because completion of EBP not only significantly reduces mental health symptoms [37] but also positively impacts physical health and functioning while decreasing PTSD-related health care costs [38,39], enhancing veteran engagement is critical. Indeed, in one study, the original CALM program was not only found to be acceptable to both providers and patients but also resulted in substantial treatment engagement and homework compliance [20].

CALM has the added benefit of being both patient and provider facing; thus, providers—not patients—directly interact with the program. The use of CALM during treatment sessions does not require patients to possess either technological proficiency or access to the internet at home. Future research could explore whether the program helps older patients gain a sense of familiarity with the use of technology in health care, thus, reducing the potential for a deepening digital divide among older adults.

Feedback, comments, and suggestions obtained from the 4 stakeholder groups, in tandem with results from similar studies [40-44], suggest a few general principles for developing or enhancing the acceptability of technology-based interventions in general. Computer- and Web-based programs should use text judiciously to reduce the potential for boredom and fatigue from reading large amounts of information [40,41]. Additionally, images and illustrations should be up-to-date [40,41], and health information should be tailored to the target audience and individualized to the patients to maximize the effect of technology-based interventions on behavior change [42,43]. Mental health interventions that receive support from providers enhance patients’ willingness to initiate computer- and internet-based treatments [44]. Because CALM is both provider and patient facing, provider support for the intervention is implicit. Patient-facing programs, however, will likely need providers to encourage their patients to initiate technology-delivered EBPs. Finally, adaptations should be based on feedback from key stakeholders, and program modifications should be presented to and reviewed by the same stakeholders.

The study design draws upon numerous strengths. An iterative design that included inviting the same stakeholders to participate in 3 waves of focus group discussions helped ensure the relevance and consistency of recommended modifications. The strength of this approach was reinforced by hosting the veteran focus group last, which allowed veterans to respond to the recommendations from the other stakeholder groups. Additionally, collecting feedback from stakeholders who are familiar with rural veterans, EBPs, and clinical practice at VA CBOCs may have increased the acceptability and feasibility of the modified program for use in this population. This approach was aligned with a person-based approach for developing and tailoring technology-based interventions [45]. Finally, using a rapid analytic technique allowed the study team to economically communicate stakeholder recommendations to the design team, ensuring that modifications were made within project time constraints.

### Limitations

One limitation of our study was that focus group participation declined throughout the study, particularly among veteran stakeholders. Because we were unable to obtain feedback from every stakeholder who participated in the first wave of focus group discussions, it is possible that we may have omitted or failed to implement suggestions as originally envisioned. Additionally, veteran and mental health provider stakeholders were recruited from one geographical region in the southern United States (Arkansas, Louisiana, and Texas). Thus, stakeholder feedback may not be generalizable to other locations. Although a necessary first step in this direction, this study also does not provide data regarding fidelity to the CBT model or treatment outcomes. A next step in this line of research is to determine whether the modified version of CALM improves VA CBOC mental health providers’ fidelity to the CBT model and improves veteran outcomes. The evidence already indicates that the original CALM program achieves these objectives [18,20]; thus, it is important to ascertain whether this has been maintained (or perhaps even enhanced) following revisions. It will also be important to assess whether implementing the program helps ensure the translation of CALM into routine clinical practice in the future because it may increase veterans’ access to EBPs at VA CBOCs.

### Conclusions

We modified the CALM program for use in rural VA clinics based on feedback from 2 waves of focus group discussions with 4 key stakeholder groups. The results of pilot testing the modified program during a third and final wave suggest that the adaptations increased the relevance and acceptability of CALM content for rural veterans and other key VA stakeholders, such as mental health providers at VA CBOCs. It will be important to assess whether using the program as an interface between providers and patients during sessions enhances veterans’ engagement in EBPs. The iterative approach used in this study holds promise for economically gathering actionable recommendations from stakeholders to enhance the acceptability and feasibility of computer-based programs in health care settings.

## Abbreviations

CALM: Coordinated Anxiety Learning Management
CBOC: community-based outpatient clinics
CBT: cognitive behavioral therapy
EBP: evidence-based psychotherapies
GAD: generalized anxiety disorder
IDT: instructional design and technology
PD: panic disorder
PTSD: posttraumatic stress disorder
SAD: social anxiety disorder
VA: US Department of Veterans Affairs

## References

[1] Cape J, Whittington C, Buszewicz M, Wallace P, Underwood L (2010). Brief psychological therapies for anxiety and depression in primary care: meta-analysis and meta-regression. BMC Med.

[2] Høifødt RS, Strøm C, Kolstrup N, Eisemann M, Waterloo K (2011). Effectiveness of cognitive behavioural therapy in primary health care: a review. Fam Pract.

[3] Leon AC, Olfson M, Broadhead WE, Barrett JE, Blacklow RS, Keller MB, Higgins ES, Weissman MM (1995). Prevalence of mental disorders in primary care. Implications for screening. Arch Fam Med.

[4] Ansseau M, Dierick M, Buntinkx F, Cnockaert P, De Smedt J, Van Den Haute M, Vander Mijnsbrugge D (2004). High prevalence of mental disorders in primary care. J Affect Disord.

[5] Kroenke K, Spitzer RL, Williams JBW, Monahan PO, Löwe B (2007). Anxiety disorders in primary care: prevalence, impairment, comorbidity, and detection. Ann Intern Med.

[6] US Department of Veterans Affairs (2008). Uniform Mental Health Services in VA Medical Centers and Clinics. VHA Handbook 1160.01.

[7] Karlin BE, Ruzek JI, Chard KM, Eftekhari A, Monson CM, Hembree EA, Resick PA, Foa EB (2010). Dissemination of evidence-based psychological treatments for posttraumatic stress disorder in the Veterans Health Administration. J Trauma Stress.

[8] Sullivan G, Blevins D, Kauth MR (2008). Translating clinical training into practice in complex mental health systems: toward opening the 'black box' of implementation. Implement Sci.

[9] Kauth MR, Sullivan G, Cully J, Blevins D (2011). Facilitating practice changes in mental health clinics: A guide for implementation development in health care systems. Psychological Services.

[10] Cully JA, Jameson JP, Phillips LL, Kunik ME, Fortney JC (2010). Use of psychotherapy by rural and urban veterans. J Rural Health.

[11] Ruzek JI, Rosen RC (2009). Disseminating evidence-based treatments for PTSD in organizational settings: A high priority focus area. Behav Res Ther.

[12] Mott JM, Grubbs KM, Sansgiry S, Fortney JC, Cully JA (2015). Psychotherapy Utilization Among Rural and Urban Veterans From 2007 to 2010. J Rural Health.

[13] Forsetlund L, Bjørndal A, Rashidian A, Jamtvedt G, O'Brien MA, Wolf F, Davis D, Odgaard-Jensen J, Oxman AD (2009). Continuing education meetings and workshops: effects on professional practice and health care outcomes. Cochrane Database Syst Rev.

[14] Stetler CB, Legro MW, Wallace CM, Bowman C, Guihan M, Hagedorn H, Kimmel B, Sharp ND, Smith JL (2006). The role of formative evaluation in implementation research and the QUERI experience. J Gen Intern Med.

[15] Jerrell JM, Ridgely MS (1999). Impact of robustness of program implementation on outcomes of clients in dual diagnosis programs. Psychiatr Serv.

[16] Foa EB, Gillihan SJ, Bryant RA (2013). Challenges and Successes in Dissemination of Evidence-Based Treatments for Posttraumatic Stress: Lessons Learned From Prolonged Exposure Therapy for PTSD. Psychol Sci Public Interest.

[17] Sullivan G, Craske MG, Sherbourne C, Edlund MJ, Rose RD, Golinelli D, Chavira DA, Bystritsky A, Stein MB, Roy-Byrne PP (2007). Design of the Coordinated Anxiety Learning and Management (CALM) study: innovations in collaborative care for anxiety disorders. Gen Hosp Psychiatry.

[18] Roy-Byrne P, Craske MG, Sullivan G, Rose RD, Edlund MJ, Lang AJ, Bystritsky A, Welch SS, Chavira DA, Golinelli D, Campbell-Sills L, Sherbourne CD, Stein MB (2010). Delivery of evidence-based treatment for multiple anxiety disorders in primary care: a randomized controlled trial. JAMA.

[19] Craske MG, Stein MB, Sullivan G, Sherbourne C, Bystritsky A, Rose RD, Lang AJ, Welch S, Campbell-Sills L, Golinelli D, Roy-Byrne P (2011). Disorder-specific impact of coordinated anxiety learning and management treatment for anxiety disorders in primary care. Arch Gen Psychiatry.

[20] Craske MG, Rose RD, Lang A, Welch SS, Campbell-Sills L, Sullivan G, Sherbourne C, Bystritsky A, Stein MB, Roy-Byrne PP (2009). Computer-assisted delivery of cognitive behavioral therapy for anxiety disorders in primary-care settings. Depress Anxiety.

[21] Hilgart MM, Ritterband LM, Thorndike FP, Kinzie MB (2012). Using instructional design process to improve design and development of Internet interventions. J Med Internet Res.

[22] Reiser R, Dempsey J (2002). Trends and issues in instructional design and technology.

[23] Gagne RM, Wager WW, Golas KC, Keller JM, Russell JD (2005). Principles of instructional design, 5th edition. Nonprofit Management Leadership.

[24] Molenda M (2003). In search of the elusive ADDIE model. Perf. Improv.

[25] Bernard H (2002). Research methods in anthropology: Qualitative and quantitative approaches. Third Edition.

[26] Sobo EJ, Seid M, Reyes GL (2006). Parent-identified barriers to pediatric health care: a process-oriented model. Health Serv Res.

[27] Hamilton A US Department of Veterans Affairs.

[28] Richards D, Richardson T (2012). Computer-based psychological treatments for depression: a systematic review and meta-analysis. Clin Psychol Rev.

[29] National CFP About Face.

[30] Kuehn BM (2012). Veterans health system cited by experts as a model for patient-centered care. JAMA.

[31] Perlin JB, Kolodner RM, Roswell RH (2004). The Veterans Health Administration: quality, value, accountability, and information as transforming strategies for patient-centered care. Am J Manag Care.

[32] McMillan SS, Kendall E, Sav A, King MA, Whitty JA, Kelly F, Wheeler AJ (2013). Patient-centered approaches to health care: a systematic review of randomized controlled trials. Med Care Res Rev.

[33] Rathert C, Wyrwich MD, Boren SA (2013). Patient-centered care and outcomes: a systematic review of the literature. Med Care Res Rev.

[34] Bertakis KD, Azari R (2011). Patient-centered care is associated with decreased health care utilization. J Am Board Fam Med.

[35] Mott JM, Mondragon S, Hundt NE, Beason-Smith M, Grady RH, Teng EJ (2014). Characteristics of U.S. veterans who begin and complete prolonged exposure and cognitive processing therapy for PTSD. J Trauma Stress.

[36] Kehle-Forbes SM, Meis LA, Spoont MR, Polusny MA (2016). Treatment initiation and dropout from prolonged exposure and cognitive processing therapy in a VA outpatient clinic. Psychol Trauma.

[37] Tuerk PW, Wangelin B, Rauch SAM, Dismuke CE, Yoder M, Myrick H, Eftekhari A, Acierno R (2013). Health service utilization before and after evidence-based treatment for PTSD. Psychol Serv.

[38] Meyers LL, Strom TQ, Leskela J, Thuras P, Kehle-Forbes SM, Curry KT (2013). Service utilization following participation in cognitive processing therapy or prolonged exposure therapy for post-traumatic stress disorder. Mil Med.

[39] Rauch SAM, Grunfeld TEE, Yadin E, Cahill SP, Hembree E, Foa EB (2009). Changes in reported physical health symptoms and social function with prolonged exposure therapy for chronic posttraumatic stress disorder. Depress Anxiety.

[40] Cucciare MA, Jamison AL, Combs AS, Joshi G, Cheung RC, Rongey C, Huggins J, Humphreys K (2017). Adapting a computer-delivered brief alcohol intervention for veterans with Hepatitis C. Inform Health Soc Care.

[41] Batterham PJ, Calear AL (2017). Preferences for Internet-Based Mental Health Interventions in an Adult Online Sample: Findings From an Online Community Survey. JMIR Ment Health.

[42] Kinzie MB (2005). Instructional design strategies for health behavior change. Patient Educ Couns.

[43] Morrison L, Moss-Morris R, Michie S, Yardley L (2014). Optimizing engagement with Internet-based health behaviour change interventions: comparison of self-assessment with and without tailored feedback using a mixed methods approach. Br J Health Psychol.

[44] Lapham GT, Hawkins EJ, Chavez LJ, Achtmeyer CE, Williams EC, Thomas RM, Ludman EJ, Kypri K, Hunt SC, Bradley KA (2012). Feedback from recently returned veterans on an anonymous web-based brief alcohol intervention. Addict Sci Clin Pract.

[45] Yardley L, Morrison L, Bradbury K, Muller I (2015). The person-based approach to intervention development: application to digital health-related behavior change interventions. J Med Internet Res.

